# Windows .NET Network Distributed Basic Local Alignment Search Toolkit (W.ND-BLAST)

**DOI:** 10.1186/1471-2105-6-93

**Published:** 2005-04-08

**Authors:** Scot E Dowd, Joaquin Zaragoza, Javier R Rodriguez, Melvin J Oliver, Paxton R Payton

**Affiliations:** 1 Livestock Issues Research Unit, Agriculture Research Service, USDA, Lubbock, TX, USA; 2 Plant Stress and Germplasm Development Research Unit, Agriculture Research Service, USDA, Lubbock, TX, USA

## Abstract

**Background:**

BLAST is one of the most common and useful tools for Genetic Research. This paper describes a software application we have termed Windows .NET Distributed Basic Local Alignment Search Toolkit (W.ND-BLAST), which enhances the BLAST utility by improving usability, fault recovery, and scalability in a Windows desktop environment. Our goal was to develop an easy to use, fault tolerant, high-throughput BLAST solution that incorporates a comprehensive BLAST result viewer with curation and annotation functionality.

**Results:**

W.ND-BLAST is a comprehensive Windows-based software toolkit that targets researchers, including those with minimal computer skills, and provides the ability increase the performance of BLAST by distributing BLAST queries to any number of Windows based machines across local area networks (LAN). W.ND-BLAST provides intuitive Graphic User Interfaces (GUI) for BLAST database creation, BLAST execution, BLAST output evaluation and BLAST result exportation. This software also provides several layers of fault tolerance and fault recovery to prevent loss of data if nodes or master machines fail. This paper lays out the functionality of W.ND-BLAST. W.ND-BLAST displays close to 100% performance efficiency when distributing tasks to 12 remote computers of the same performance class. A high throughput BLAST job which took 662.68 minutes (11 hours) on one average machine was completed in 44.97 minutes when distributed to 17 nodes, which included lower performance class machines. Finally, there is a comprehensive high-throughput BLAST Output Viewer (BOV) and Annotation Engine components, which provides comprehensive exportation of BLAST hits to text files, annotated fasta files, tables, or association files.

**Conclusion:**

W.ND-BLAST provides an interactive tool that allows scientists to easily utilizing their available computing resources for high throughput and comprehensive sequence analyses. The install package for W.ND-BLAST is freely downloadable from . With registration the software is free, installation, networking, and usage instructions are provided as well as a support forum.

## Background

### What is BLAST?

Basic Local Alignment Search Tool (BLAST) answers the question: "What known nucleotide or amino acid sequence in an existing sequence database is most similar to an unknown input (query) sequence?" BLAST was originally intended for comparison of unknown and newly sequenced genetic sequences against annotated database sequences to find those sequences with the closest biological similarity to the query [1,2]. Many papers describe the BLAST algorithm and concepts for similarity searching in detail [1-3].

With the advent of high-throughput sequencing technologies, there is an ever increasing input of new genetic data, which typically needs to be evaluated using BLAST. As this ever increasing volume of new data is analyzed using BLAST and then annotated the new sequences are subsequently added to the public databases, which in turn continues to increase their size. This is a vicious cycle, in which new sequence data is generated at increasing rates and databases are continually increasing in size. The BLAST search process is therefore becoming an increasingly time-consuming productivity bottleneck in genomics. In example, a typical laboratory workstation (2.4 Ghz Pentium 4 processor, 1 GB physical memory, 60 GB SATA hard drive) can search one query sequence (800 bp) against a local copy of NCBI  nucleotide (nt) database (320 million bp database) in just under 3 minutes. Thus, a typical high-throughput BLAST search, which involves 10,000 sequences, can take over 20 days to complete on a single workstation. This is an inordinate amount of time to wait for results.

There is a growing need to find ways of reducing these BLAST productivity bottlenecks when evaluating new sequence data. One of the most logical approaches is to increase overall BLAST performance by enhancing scalability, user-friendliness, and reliability. There are an increasing number of commercial and several non-commercial software packages that have been developed (Table 1). The obvious drawback to commercial products is a very high price tag. On the other hand, non-commercial and freely available BLAST solutions (Table 1) have been shown to increase the throughput of large BLAST jobs, but typically require a high level of computer literacy and networking skill to enable them to be effectively utilized. As examples, WU-BLAST [3] provides a dramatic increase in the speed and efficiency of the BLAST algorithm itself, but it is currently unavailable for Windows environments, does not have distribution capabilities inherent, and requires someone with above average computer skill to setup, operate and maintain. Similarly, MPI-BLAST [4], which is arguably the most popular and powerful distributed BLAST implementation available today, distributes BLAST across Windows, UNIX-based, or heterogeneous networks, yet need for extensive networking skill, lack of user interfaces, and low fault tolerance can result in limited utility for the average end-user.

Ultimately the high price, lack of user-friendliness, or other similar factor reduces the utility of most available technologies for a majority of scientists that could truly benefit from such high-throughput BLAST tools. Thus, after evaluating the available free software, we identified the need within the research community for a comprehensive BLAST toolkit with a high degree of user-friendliness and functionality. We sought to create a full Windows toolkit with the following characteristics:

• Utilizes the most common operating system (Windows 2000 and Windows XP)

• Utilizes Local Area Network (LAN) connected Windows workstations as a distributed environment

• Freely available for non-commercial and academic use.

• Does not require extensive networking, hardware or software skills to setup and utilize.

• GUI based formatting of custom databases from any fasta file

• GUI based importing and distribution of existing BLAST databases (architecture dependent)

• Automatic scanning of LANs for Windows workstations that are available and ready to run WND-BLAST

• Ability for users at child nodes to cancel computationally intensive BLAST searches without disrupting the entire job if they happen to require more resources than are available during searches.

• Ability to BLAST a multi-FASTA file (single file containing more than one fasta sequence)

• Ability to BLAST a folder containing multiple single fasta files (single folder with multiple files)

• Automatic creation and management of individual projects on any workstation in the network.

• Generation and real-time viewing of BLAST log files to show network performance and progress

• Progress bars for most time consuming functions such as database formatting and large high throughput BLAST searches

• Logical formatting and visualization of BLAST search outputs using any of the fields available (e.g. bit score, hit def, E-value etc.)

• Exporting of custom annotated FASTA files containing any of the BLAST output data fields or manually input data.

• Exporting of fasta files containing any combination of hits, "no hits", or "false hits".

• Ability to manually and automatically curate BLAST output files

• Indexing of input and output files related to annotation allowing for rapid database curation and intuitive manipulation and mapping of data along with other experiments.

• GUI based installation and easy to follow instructions for setting up required Windows network shares.

• A useful help file.

The solution developed to address all of these issues has been named W.ND-BLAST, which stands for Windows .NET Distributed Basic Local Alignment Search Toolkit.

## Implementation

### Programming language

The package was written using Microsoft Visual Studio .NET 2003 and C# as the primary programming language. The software relies on the use of .NET 1.1 framework, which can be freely downloaded from the Microsoft website .

### Auxiliary programs

W.ND-BLAST has encapsulated the NCBI BLAST software  including blastall, formatdb and scoring matrices (i.e. Blosum62, PAM). W.ND-BLAST only requires a user supplied database and input sequences for proper execution. W.ND-BLAST has the ability to execute any of the sub-programs of blastall (i.e. blastx, tblastx, blastn etc).

### W.ND-BLAST Toolkit overview

The W.ND-BLAST toolkit consists of several integrated software solutions

• Project Control Panel

• Database Engine

• BLAST Engine

• W.ND BLAST Distribution Algorithm

• BLAST Output Viewer

• Annotation Engine

#### Project Control Panel

**Figure 1:**
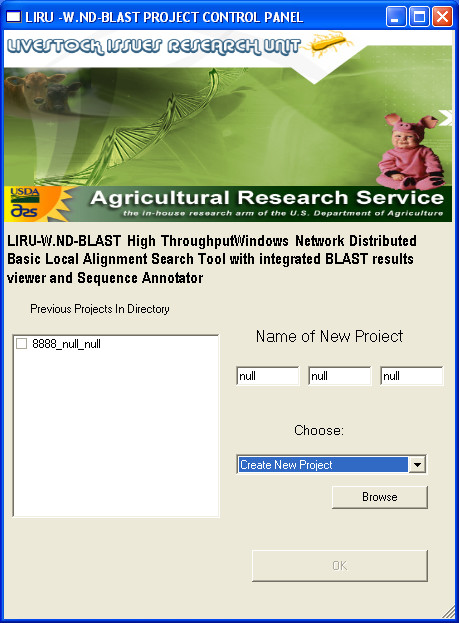
**Screenshot: Project Control Panel (PCP)**. The Project Control Panel (PCP) displays the user's options for W.ND BLAST. This example screenshot shows where the user is able to "Create New Project" by simply entering in a new project name. The "Create New Project" can also be changed to "Load Saved Project" where the user has the option to choose a previous project from the left text box.

**Figure 2:**
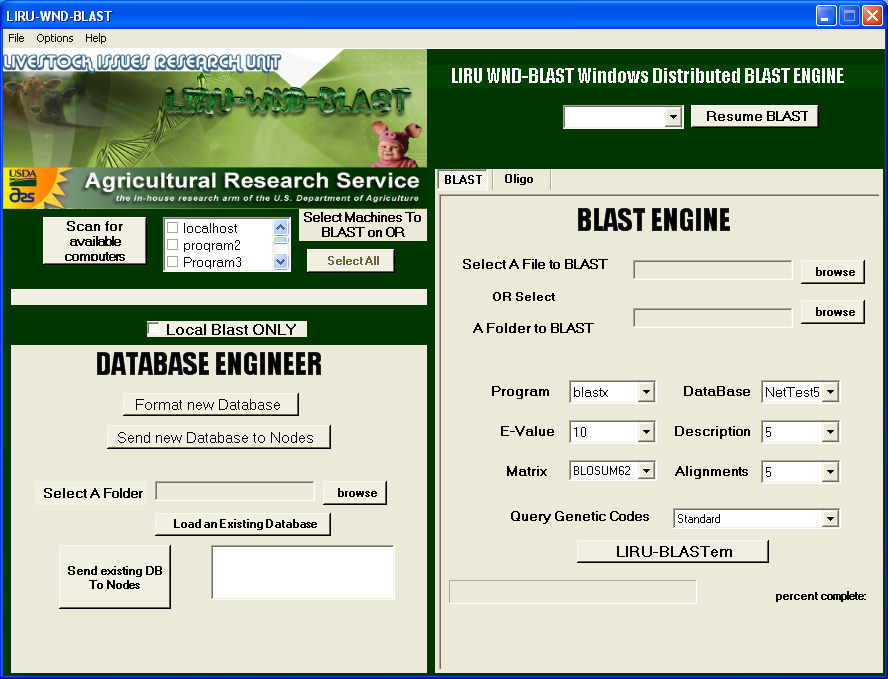
**Screenshot: Database and BLAST Engine (DBE)**. This example screenshot displays the Database Engineer, which is used for scanning for child nodes, creating custom databases and distributing existing databases. Within the same interface is the BLAST engine, which is used for executing inputting query sequences, choosing BLAST parameters, and executing BLAST jobs. Also available is the ability to control child nodes during BLAST jobs. Lastly, the user has the option of resuming a failed BLAST.

**Figure 3:**
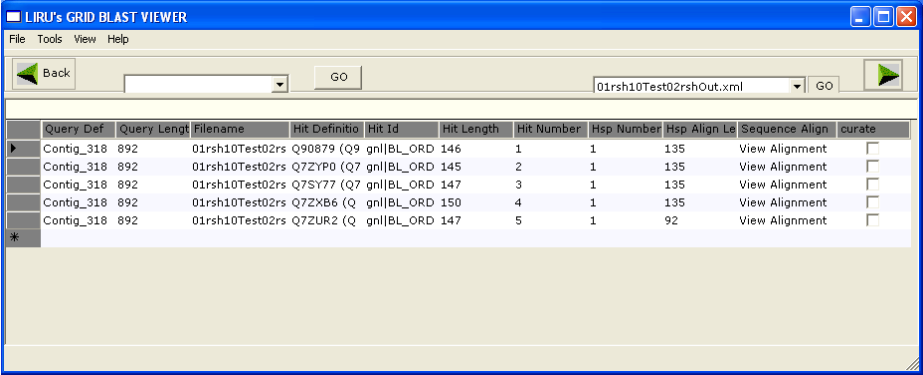
**Screenshot: BLAST Output Viewer (BOV)**. This example screenshot of the BLAST Output Viewer displays some of the available columns information from an individual BLAST hit. This particular window is displaying the first five hits for a sequence named, "Contig_318." From here the user can move forward or backward through all BLAST output sequences. The user can also click on the "View Alignment" column to bring up the "Sequence Alignment Viewer" window.

Considerable attention was put into the design of the W.ND-BLAST protocol, which is based on creation and organization of separate BLAST projects. Once the software is initiated, the user is presented with a simple Project Control Panel (PCP) (Figure 1). This control panel has two options: A) create a new project or B) work on a previous project. The user creates a new project by entering the investigators last name, the title of the project, and a database identifier or date. After the researcher defines the new job the combined Database and BLAST Engine (DBE) interface opens and a unique project folder is automatically created for the new project (Figure 2). Alternatively the second option available at the (PCP) is to work on an existing or previous project. If an existing project is selected the BLAST Output Viewer (BOV) is opened along with a list of available subprojects (Figure 3). Subprojects can represent multiple BLAST outputs of the data against different or updated databases or sub-queries from the input sequences.

#### Database Engine

The Database Engine is a separate part of the previously mentioned DBE (Figure 2), which allows for creation and distribution of new databases using a fully functional database formating (formatdb) interface. The formatdb interface provides all available parameters with the most common parameters defaulted. Thus, when formatting a new database only the location of the fasta file being formatted must be identified (using a browse feature) and the type of fasta file (protein or nucleotide) must be entered. The database is formatted and entered into the software appearing as an available database. Any number or type of database can be created and made available. The Database Engine also allows for automatic location and importing of existing databases, which are copied into the database (DB) directory and added to the available database file. The software also has the ability to scan a computer or specific folder and will find any existing databases. Following scanning for available workstations in the next section, the databases can be automatically distributed to any or all of the worker nodes after formatting or importing.

#### BLAST Engine

The BLAST Engine, which is the primary part of the DBE (Figure 2), provides the option to scan the LAN for available network workstations (nodes) running the W.ND-BLAST server. It also provides the alternate option to BLAST only on the local machine. The software scans and finds only those LAN workstations that are available and configured to run WND-BLAST. The same WND-BLAST package provided for the master node software must be installed on each of the worker nodes as well. The BLAST Engine allows for the selection of child nodes, fasta file or folder, and BLAST parameters. The user is also presented with a list of BLAST databases to choose. Note: the user must format or import these databases. Progress bars keep track of the job and a real-time log file viewer provides feedback to the user on the overall progress of the BLAST as well as on which workstations are working.

#### W.ND BLAST distribution algorithm

The software utilizes simple client/server architecture with a single source client, referred to as 'master node' and multiple servers that are referred to as 'child nodes'. An included W.ND-BLAST server must be installed and running on all participating child nodes. Each child node is used solely for communication with the master node and executing the BLAST program. The BLAST algorithm used is unchanged from the most current NCBI BLAST, version 2.2.9. As newer versions are released by NCBI the algorithm can be updated independently with an integrated update feature or with new versions of W.ND-BLAST provided through the Livestock Issues Research Unit's website. The child nodes are queried by the master node upon execution of the BLAST algorithm. Communication between the master and child nodes is achieved and maintained through a well known port using an underlying Transmission Control Protocol (TCP) connection.

The child nodes are located by W.ND-BLAST, which performs a search of all workstations within range (e.g. domain or subnet mask) of the master or local machine. This is accomplished by creating a unique multicast group for the master and child nodes. Once communication has been established by the master node, the child nodes are added to the *available workstation pool*. The user is given a choice of using all or any combination of child nodes in a pool for their job. Thereafter, the *available child node pool *can be used to add child nodes, while the BLAST is in progress. This is useful when a user knows his colleagues no longer need their compute resources and wishes to add this newly freed resource to his currently running BLAST job. Before the master node executes a BLAST query, each participating child node must have the database the user wishes to query available locally. If not available locally, the master will distribute the database to the child nodes lacking the database. Once each child node has the required database, the master begins running queries on that child.

To add flexibility to input data processing, the user has the option to select a single multi-fasta file or an entire folder containing individual fasta files. If a multi-fasta file is selected, W.ND-BLAST automatically parses the file into single fasta files. If a whole folder is selected, W.ND-BLAST will attempt to BLAST each fasta file contained in that folder, but each file in the directory must only contain a single sequence. Once the user identifies either a file or folder every sequence in the pool is marked as *not done *in the master node job log at initialization of the "BLASTem" command. The sequences are then marshalled and sent one by one to child nodes. The master log reflects this distribution by updating the status of each sequence to *working*. Upon receipt, the child node un-marshals the query and begins BLAST operations. After the initial sequence distribution to each node, the master node is placed in a wait state tied to that sequence. The master node remains in this state until it receives a call-back from one of the child nodes or there are zero sequences remaining in its waiting sequence pool. Every child node, upon completion of a BLAST, sends a call-back message to the master informing of its finished state. The master node then retrieves the BLAST results and writes them to a file in the project folder. Upon receipt of the call-back, the master node also sends the child node the next query sequence from the pool to begin BLAST operations and update the sequence status to *done*. This process continues for every sequence until the sequence pool is empty.

#### BLAST Output Viewer

**Figure 4:**
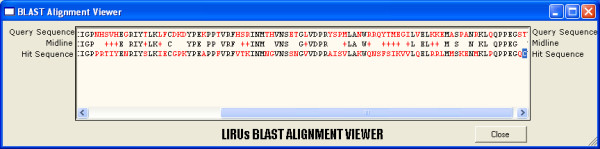
**Screenshot: Sequence Alignment Viewer**. This screenshot of the Sequence Alignment Viewer gives an example the BLAST output for one alignment. The query sequence, midline and hit sequence are displayed in parallel and pair-wise equal can be seen in black

Once a BLAST is completed, the user has the option to view the BLAST output in the BLAST Output Viewer (BOV, Figure 3). The viewer scans through each BLAST output, one input sequence at a time, and displays as many hits for each sequence as the user desires. The viewer displays hits in a tabular format that can be rearranged based upon the user's desired preference. The viewer works with any type of XML formatted BLAST output such as BLASTx, BLASTn, BLASTp, tBLASTx, etc. There is also an alignment option that brings up a separate window that displays any of the output alignments. The alignment view of the sequence shows the query-seq, hit-seq, and mid-line fields of the hit aligned for easy viewing and analysis (Figure 4). The curation of BLAST hits is performed by clicking the checkbox corresponding to a single BLAST hit. This manual curation is based on which hits the user decides are significant based on his/her criteria. The researchers' criteria may consist of bit-score, e-value, and hit length for example. Curated hits can be exported as tables, association files and even annotated fasta sequences.

#### Annotation Engine

**Figure 5:**
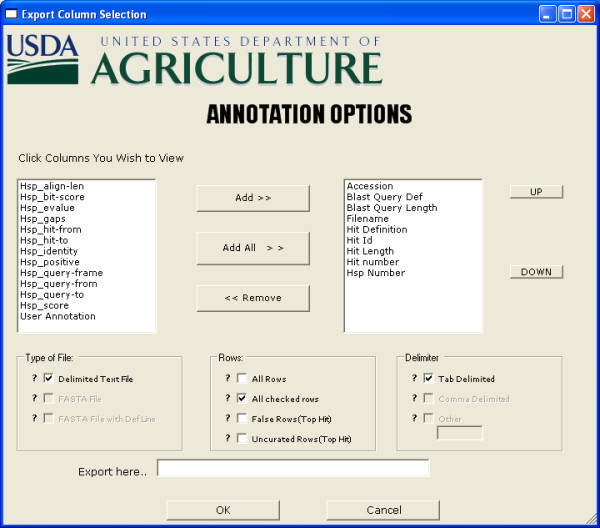
**Screenshot: Annotation Engine**. The Annotation Engine window shown here gives the user the options to export their data in several different formats. The user can choose from any of the available BLAST output result fields and any available type of output file and associated delimiters.

The BOV acts not only as a way to view and curate high-throughput BLAST data, but also has an effective way to perform automated annotations based upon top hits. The type of annotation can be defined dynamically based upon hit definition and can include any of the BLAST output fields that are available. The Annotation Engine (Figure 5) is used to export the BLAST output data according to the user's specifications. The Annotation Engine can automatically create annotated multi-fasta files, tab delimited text files containing all annotation, or common association files that are fully annotated based upon the user's criteria or selections.

In addition, as one of the most powerful aids to downstream analysis, the software automatically creates separate FASTA file folders within a project that contain the "no hits". This allows the "no-hits" from previous jobs to be queried against separate databases or future updated database versions. Similarly, during manual curation (as described above), those hits not "curated" or considered significant by the scientist are considered "false hits". As with the "no-hit" results the "false-hit" files can be exported separately as fasta files. As indicated, no-hits are those queries which did not get a significant match against the database.

An example of a typical annotated fasta output of an unknown sequence (contig 111) searched against a typical protein databases that hit against the database sequence with accession number Q143235 could be exported as follows (the following sequence is for demonstration only): >contig 111- similar to GB:AF166120.1 -E-value 1 × 10e-180- transcription activator AAGCTTCACGCCCAATTCTGCATCATTTTCATAAAGAGCAGGATTGGACAATACTTGAAAACTCAGTTTATTGGCTGCGTCGGAGGCGTTGGAGATCAACTCACGTAGG...

### Workstation test network

The workstation test network used for testing was limited to the number of workstations available within our facility (25 windows workstations). All of the workstations have various versions of Microsoft Windows (e.g. XP, 2000, 2003 server, and 98). The workstations used in the testing process are detailed in Table 2.

### Performance testing

Various combinations of workstations from the test network were utilized to test the functionality and reliability of W.ND-BLAST. For performance tests, the job start times were automatically recorded as well as the time when the job finished. Duration of the job was calculated by subtracting the software recorded initiation time from the software recorded end time. Performance of W.ND-BLAST was tested by varying the number of input sequences, database size, and number of workstations. Two sizes of databases were used for testing the functionality of W.ND-BLAST, a 332 MB (total formatted size) protein (able to reside in physical memory) and 1.5 GB (total formatted size) nucleotide database (too large to reside in physical memory). The primary rational for testing the larger database was that the larger database exceeds the caching ability of the physical memory and requires the computer to access disk. In addition, larger databases are automatically segmented by formatdb. For this reason it is necessary to test both the database engine and BLAST engine for their abilities to handle large and segmented databases. The current version of W.ND-BLAST improves BLAST scalability only by distributing jobs across networks. As noted in the future work section at the end of the manuscript future versions of W.ND-BLAST will be further optimized to enhance performance on increasingly large databases using database partitioning (segmentation) and dynamic task allocations (Quality of Service partitioning). The smaller database was populated by protein sequences so BLASTx was executed. The larger database was populated by a subset of nucleotides from NCBI's nt database, therefore, BLASTn was executed. Current versions of NCBI nt and NCBI nr databases were tested to ensure that the software was able to handle much larger databases. Finally, all of the common BLAST programs were tested (BLASTp, BLASTx, BLASTn, and tBLASTx) and found to perform correctly.

### Fault tolerance/recovery implementation

As with any distributed system, fault tolerance is always a key issue. W.ND-BLAST displays several layers of fault tolerance. The primary level of fault tolerance is achieved by allowing the master node to assign individual and distinct jobs to each child node. Because jobs (each individual sequences) are submitted independently by the master node to each child node, if a node fails, only the single sequence the failed node was processing at the time of failure is temporarily lost. The lost sequence is ultimately reallocated to a separate node by the master so that there will be no missing data. A log is kept at the master node of all of the queries in the sequence pool (jobs waiting to be executed) and their respective status. When a result is received at the master node, the validity of the result is checked, the sequence status is changed to *done *in the log, and the sequence is removed from the pool. The master node will only allow the sequence to remain in the *working *state for a user specified amount of time before returning the status to *not done*. This prevents jobs from being stalled on slow nodes toward the end of large jobs. When a failed child node is restarted on the network, it will broadcast a message to the master informing it of its return to *ready *status. The master then prompts the user to add or omit this node to its *working nodes*. If the master node goes down (upon rebooting of the master node or restart of the software), the job can be resumed at the point of failure and all data up to that point is maintained intact. All remaining query sequences in the sequence pool will then resume execution on the available children. The final layer of fault tolerance occurs at the end of a large BLAST job when the software performs a final check of data integrity and ensures that all the input sequences have generated a quality output. Any sequence that did not generate an output file is automatically subjected to a second BLAST.

### Efficiency

Efficiency (calculated as a percentage) was used in order to determine the scalability of W.ND-BLAST. Efficiency was calculated by means of the following equation:

E = [σ / (n * x)]*100%.

where:

E = efficiency expressed as a percentage.

σ = Time to process a given number of sequences (job) on a single average class E (Table 2) workstation.

n = Number of workstations being evaluated for a given data point (e.g. if 9 workstations are being tested n = 9). This does not include the master node which does not perform queries.

x = Time to process job on n workstations.

## Results and discussion

### Performance of W.ND BLAST

**Figure 6:**
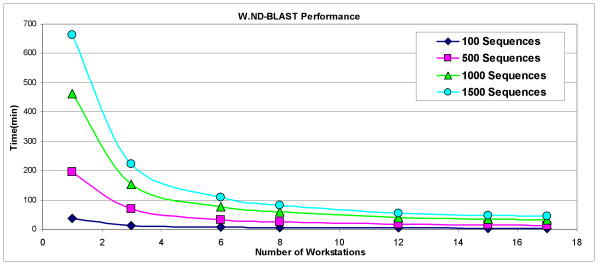
**WND-BLAST Performance**. This graph presents the performance of WND-BLAST observed when varying the number of workstations and input sequences. To collect this data, we utilized W.ND-BLAST to perform BLASTx against a 332 MB (formatted size) database with the varying number of input (query) sequences shown.

**Figure 7:**
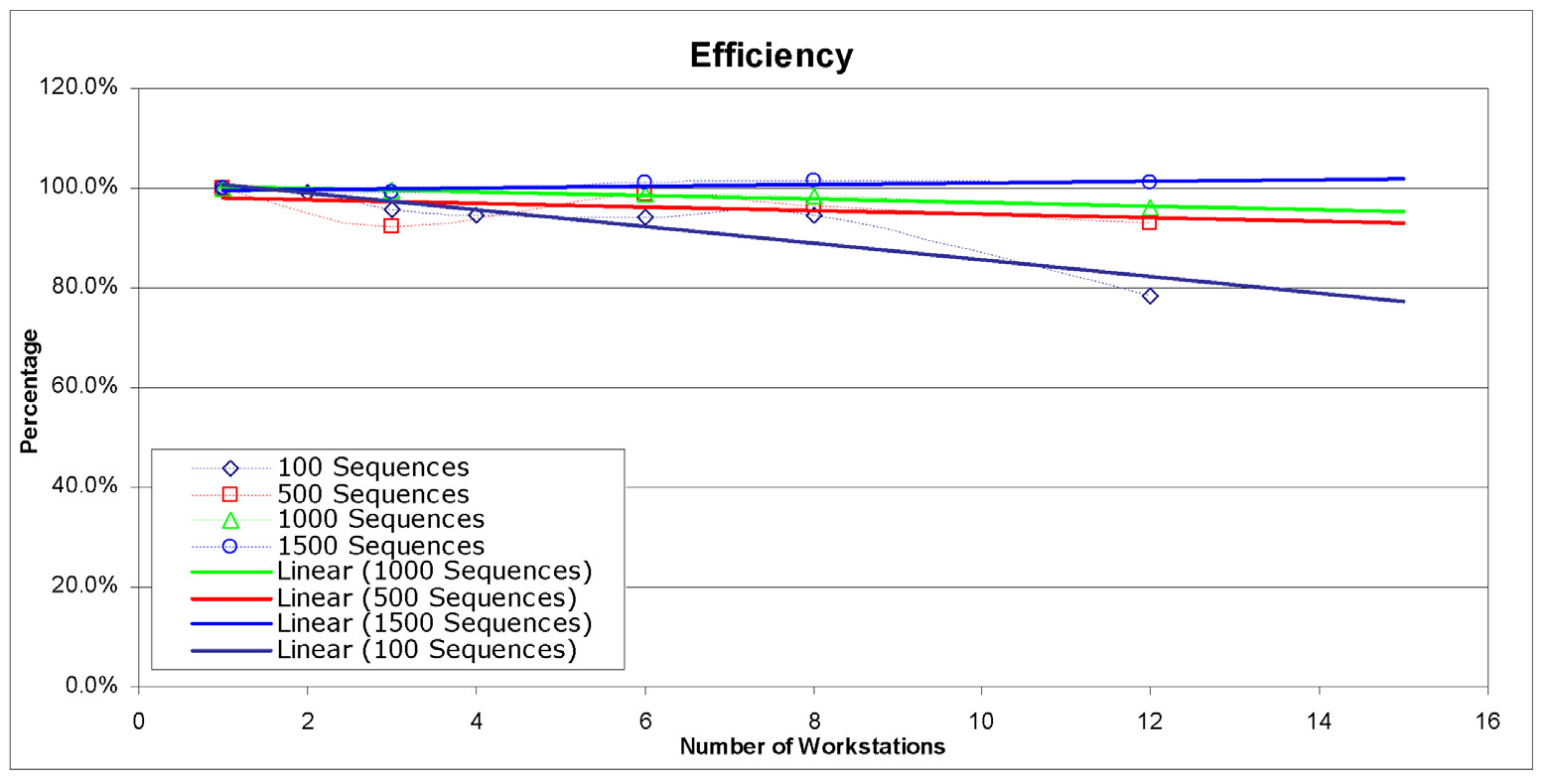
**Workstation Efficiency Percentage with Best Fit trend lines**. This graph shows the overall efficiency of performance of the data presented in Figure 1, as the number of machines is increased. Equation 1 from the text is used to generate this data. The linear best fit regressions for the data points are also provided.

Figure 6 provides data to illustrate the time savings realized with use of W.ND-BLAST on increasing numbers of workstations. This data was obtained by performing BLASTx for various numbers of sequences against a 332 MB (formatted size) database. The figure indicates a decrease in the amount of time required for BLAST of large input files as the number of workstations is increased. W.ND-BLAST exhibits an almost inverse linear relationship between the time taken to complete a given job and the number of workstations utilized for that job. This trend is very evident when considering Figure 7, which displays the efficiency of W.ND-BLAST as additional machines are added. Note that the number of workstations was limited to available workstations within our laboratory and we cannot project how network performance would affect this linear relationship when increasingly large numbers of workstations are utilized. Table 3 provides the raw data for the completion of test jobs as time in minutes.

When performing the test using 1500 sequences on one workstation the BLASTx took on average 662.68 min. Theoretically, when increasing the number of workstations by three the time should decrease by no more than 3 times (220.89 minutes). The actual time, derived from Table 3, was measured at 222.67 minutes, which correspond to a 99% efficiency rating using Equation 1. With 6 workstations the time should theoretically be 110.33 minutes. The actual average time achieved was 109.12 minutes, which gives an efficiency rating of better than 100%. This trend can also be seen when doubling the number of workstations from 6 to 12. It is likely that those efficiencies above 100% are likely attributed to the effect of adding worker nodes with slightly more resources (e.g. Class D) as part of the child node pool. This is because efficiency is calculated in relation to the single machine times, which are determined on our Class E nodes (Table 2).

Figure 7 exhibits the sequence sample size efficiency expressed as a percentage (Equation 1) when plotted for all benchmarks. These data demonstrate that W.ND-BLAST is more efficient when using a larger number of query sequences. The efficiencies for 15 and 17 workstations were not calculated because experiments were forced to be performed on workstations of much lower (Class F – I) or of much higher compute performance capabilities (Class A). However, the results using these additional workstations illustrate scalability and are provided in Table 3. Table 4 displays the results of BLAST scalability using even larger databases (1.5 GB) indicating that W.ND-BLAST can accommodate and make more efficient such intensive database queries. As a final operability test we obtained and tested W.ND-BLAST on 12-15-04 full and segmented versions of both NCBI nr and nt databases and found it was easily able to utilize even very large databases (data not shown).

### Fault tolerance/recovery

W.ND-BLAST allowed workstations to fail, be turned off, or to be turned on during jobs. It easily adapted by redirecting failed queries, adding new workstations, or ignoring failed workstations. Even when the master node was shut off during testing and although the job did not progress, W.ND-BLAST was able to continue the job at the point of failure once the master node was rebooted.

### Shortcomings in current version

W.ND-BLAST was designed as a user-friendly distributed software implementation of BLAST. For large databases such as current versions of nr or nt from NCBI there is a inherent decrease in the efficiency of the BLAST algorithm as described in the introduction. By distributing tasks among computers W.ND-BLAST still reduces the time it takes to perform searches on any size databases (in proportion to the number of worker nodes utilized) but does not enhance the efficiency of the BLAST algorithm itself. With tools such as MPI-BLAST [4], which utilizes a database segmentation scheme, queries against large databases are more efficient. As noted in the following section on future work the next evolution of W.ND-BLAST will include a database segmentation scheme that will dramatically improve overall efficiency on increasingly large databases.

In the current version of W.ND-BLAST there is the inability of the software to handle multiple projects simultaneously originating from a single master node. However on large networks multiple instances of the software can be running simultaneously if separate master processes are activated from different workstations and worker nodes are partitioned between jobs. This potentially allows more than one scientist to be utilizing the software on the same network.

W.ND-BLAST software is able to do a BLAST locally on the master node alone (local BLAST) but it is unable to BLAST on the master node, while performing distributed BLAST. This means that the number of machines for distributed BLAST is always n-1 if the master node is counted. It should be noted that all calculations based on time in this paper do not count the master node. In W.ND-BLAST the master node distributes tasks, coordinates, and compiles results.

Finally, there is a lack of ability in the current version to utilize more than one processor on multi-processor machines. In future versions this feature will be reincorporated as a feature.

### Future work

There are several areas where W.ND-BLAST would benefit from future work. Firstly, W.ND-BLAST provides for sufficient fault tolerance when the master node fails, but implementing a shadow master would be the most efficient addition to the algorithm. The shadow master would act as a clone to the master node, and in the event of failure the shadow would continue the execution of the application. If the master node fails, the current implementation would not resume until the user arrived back at the master workstation to restart it. With a shadow master, the software would continue. Secondly, one of the most dramatic planned performance improvements in the W.ND-BLAST implementation will be the development of a database segmentation scheme similar to MPI-BLAST [4]. Considering the efficiency of W.ND-BLAST on smaller databases a segmentation scheme should provide an even greater increase in efficiency on large databases such as current versions of nr or nt. This increase in performance would be primarily based on the ability of a given node to maintain a designated segment (small piece) of large databases in its memory so that it does not exceed the physical memory capacity and allowing it to cache the database files in memory after the first search. This prevents excessive writing to disk to load additional portions of the database. Instead by segmenting the database a node will typically search all sequences against its own small piece of the database and return results to the master to be compiled. Thirdly, several levels of Quality of Service (QoS) will be implemented within the W.ND-BLAST system. The current implementation only allows the remote users (users at nodes) to end the application if necessary. Future efforts will allow the user at the worker node or at the master node to decrease the amount of CPU usage BLAST is allotted on each node on a node by node basis. QoS will also be implemented related to the availability or lack of availability of disk space on child nodes. In example, if the child node is unable to hold the database it can automatically be pointed or manually assigned to access a file share database.

## Conclusion

BLAST is one of the most widely used applications in modern biology, including genomics, microbiology, and molecular biology in general. Its significance can be shown in the thousands of publications that refer to its use every year. In science, when a tool is this significant, the next logical step is to improve its functionality (ease of use). WND-BLAST provides this with its no hassle installation and intuitive user interfaces. Also, when a tool is this widely used the need arises for enhancing performance. WND-BLAST accomplishes this by allowing the user to distribute BLAST jobs from a single workstation to all available computing resources without the need for a server class machine. WND-BLAST provides the research community with a more comprehensive, fault tolerant, user-friendly, reliable, and time efficient toolkit to perform BLAST queries distributed across Windows based networks. W.ND-BLAST's output viewer and annotator also provide the user with a high-throughput method to analyze, process, and export BLAST results in a well-organized fashion.

## Availability and Requirements

WND-BLAST can be downloaded free-of-charge from the web page . WND-BLAST requires Windows 2 k or higher with the .NET 1.1 framework installed. As mentioned previously, WND-BLAST was written primarily using Microsoft Visual Studio .NET 2003 using C#. The WND-BLAST software is provided 'as is' with no guarantee or warranty of any kind. The WND-BLAST software is available for all non-commercial use. Any other use of the software requires special permission from the primary author.

### USDA disclaimer

Mention of trade names or commercial products in this publication is solely for the purpose of providing specific information and does not imply recommendation or endorsement by the U.S. Department of Agriculture.

## Authors' contributions

SD conceived of the project, devised the W.ND-BLAST algorithm, designed the functionality of each aspect of the software, and edited very early versions and final drafts of the manuscript; JZ encoded much the software and wrote the primary version of the manuscript; MO assisted with testing, finding bugs in the software, and editing early version of the manuscript; JR assisted with all coding of the software; PP assisted in writing the manuscript and testing the software.

## Figures and Tables

**Table 1:** BLAST tool comparison

**Program Name**	**Reference**	**Increase BLAST Performance**	**Freely Available**	**Distributed BLAST**	**GUI Based**	**GUI BLAST Results Viewer**	**GUI Database Formatter**	**OS Available^1^**	**Fault Recovery^2^**
**NCBI BLAST**	1	N/A	yes	No	No	No	No	W,U	Low
**BLAST++**	5	yes	yes	No	No	No	No	W,U	Low
**WU-BLAST**	3	yes	yes	No	No	No	No	U,O	Low
**DeCypher BLAST™**	6	yes	No	No	yes	No	No	O	High
**S-BLAST**	7	yes	yes	yes	yes	No	No	W,U	High
**BeoBLAST**	8	yes	yes	yes	No	No	No	U	Medium
**StarBLAST**	9	No	No	No	yes	yes	yes	W	Low
**mpiBLAST**	4	yes	yes	yes	No	No	No	W,U	Low
**Condor BLAST**	10	yes	yes	yes	No	No	No	W,U	Medium
**Soap-HT-BLAST**	11	yes	yes	yes	yes	No	No	W,U	Medium
**Paracel**	12	yes	No	yes	yes	yes	No	U,O	Unknown
**W.ND-BLAST**	13	yes	yes	yes	yes	yes	yes	W	High

1 – W = Microsoft Windows, U = UNIX/LINUX, O = Other

2 – Based on ability to recover from node failure, master failure, and complete failure

This table provides a list of many of the most popular BLAST programs as well as their major features. The primary goal of most BLAST packages is to increase the performance of the BLAST algorithm. There seems to be a dichotomy between increased performance and user friendliness that has been encapsulated within W.ND BLAST. W = Windows based, U = Unix/Linux based, O = Other

**Table 2:** Workstation specifications

**Class**	**Processor**	**Operating System**	**Number of workstations**	**RAM**	**Harddisk Space**
**A**	Dual Xeon 3.0 Ghz	Microsoft Windows XP Pro	1	6 GB	80 GB Ultra320 SCSI RAID0
**B**	Pentium 4 Extreme 3.2 Ghz	Microsoft Windows Server 2003	1	1 GB	140 GB SATA RAID0
**C**	Pentium 4 2.8 Ghz	Microsoft Windows XP Pro	2	2 GB	40 GB SATA
**D**	Pentium 4 2.4 Ghz	Microsoft Windows XP Pro	2	2 GB	80 GB SATA
**E**	Pentium 4 2.4 Ghz	Microsoft Windows XP Pro	12	1 GB	80 GB SATA
**F**	Pentium 4 2.0 Ghz	Microsoft Windows XP Pro	1	1 GB	40 GB
**G**	Pentium 4 1.8 Ghz	Microsoft Windows 2000 Advanced Server	1	512 MB	40 GB
**H**	Pentium 4 1.4 Ghz	Microsoft Windows 2000 Pro	1	128 MB	20 GB
**I**	Pentium 3 M 1.0 Ghz	Microsoft Window 2000 Pro	1	512 MB	20 GB

Different classes of workstations used in testing W.ND-BLAST are given. They are classified according to processor type and speed, OS, RAM, and hard disk space. The number of each workstation class in our lab is also given.

**Table 3:** Performance evaluation Number of Sequences

	Number of Sequences			
	**100**	**500**	**1000**	**1500**

**Number of Workstations**	**W.ND-BLAST**	**W.ND-BLAST**	**W.ND-BLAST**	**W.ND-BLAST**

**1**	38.15	194.68	461.81	662.68
**3**	19.20	70.36	154.28	222.67
**6**	13.28	32.79	77.88	109.12
**8**	10.09	29.50	58.58	81.70
**12**	6.74	17.45	40.10	54.66
**15**	5.04	13.92	33.79	46.67
**17**	4.05	13.33	32.58	44.97

This table provides the time in minutes to perform the specified BLAST jobs on the number of sequences shown. The database size used for this analysis was 332 MB (formatted size) protein database. BLASTx was utilized to search high-throughput jobs ranging from 100–1500 sequences on various heterogenous workstations ranging from 1 to 17 nodes per job. At 15 and 17 nodes it should be noted that older machines were utilized.

**Table 4:** BLAST performance for large databases gives the average times (n = 3) for a W.ND-BLAST project to complete when querying 50 sequences on Class E machines against a 1.5 GB nucleotide database (too large to fit in physical memory). The times, shown in minutes and seconds, show a relatively large decrease in execution time when the number of machines is increased from one to seven nodes.

**Sequence Number**	**Database Size**	**Number of Machines**	**Total Time(mm:ss)**
50	1.59 GB	1	63:57
50	1.59 GB	7	12:14
